# *In vivo* Dynamic Phase-Contrast X-ray Imaging using a Compact Light Source

**DOI:** 10.1038/s41598-018-24763-8

**Published:** 2018-05-01

**Authors:** Regine Gradl, Martin Dierolf, Benedikt Günther, Lorenz Hehn, Winfried Möller, David Kutschke, Lin Yang, Martin Donnelley, Rhiannon Murrie, Alexander Erl, Tobias Stoeger, Bernhard Gleich, Klaus Achterhold, Otmar Schmid, Franz Pfeiffer, Kaye Susannah Morgan

**Affiliations:** 1 Chair of Biomedical Physics, Department of Physics, Technical University of Munich, James-Franck-Str. 1, 85748 Garching, Germany; 2 Munich School of BioEngineering, Technical University of Munich, Boltzmannstr. 11, 85748 Garching, Germany; 3 Institute for Advanced Study, Technical University of Munich, Lichtenbergstr. 2 a, 85748 Garching, Germany; 4 Max-Plank-Institute of Quantum Optics, Hans-Kopfermann-Str. 1, 85748 Garching, Germany; 5 Department of Diagnostic and Interventional Radiology, Klinikum rechts der Isar, Technical University of Munich, Ismaninger Str. 22, 81675 München, Germany; 6 Comprehensive Pneumology Center, Member of the German Center for Lung Research (DZL), Max-Lebsche-Platz 31, 81377 München, Germany; 7 Institute of Lung Biology and Disease, Helmholtz Zentrum München - German Research Center for Environmental Health, Ingolstädter Landstraße 1, 85764 Neuherberg, Germany; 8 Robinson Research Institute and Adelaide Medical School, University of Adelaide, Adelaide, Australia; 9 Respiratory and Sleep Medicine, Women’s and Children’s Hospital, 72 King William Road, North Adelaide, SA 5006 Australia; 10 Department of Mechanical and Aerospace Engineering, Monash University, Clayton, Victoria, 3800 Australia; 11 School of Physics and Astronomy, Monash University, Clayton, Victoria, 3800 Australia

## Abstract

We describe the first dynamic and the first *in vivo* X-ray imaging studies successfully performed at a laser-undulator-based compact synchrotron light source. The X-ray properties of this source enable time-sequence propagation-based X-ray phase-contrast imaging. We focus here on non-invasive imaging for respiratory treatment development and physiological understanding. In small animals, we capture the regional delivery of respiratory treatment, and two measures of respiratory health that can reveal the effectiveness of a treatment; lung motion and mucociliary clearance. The results demonstrate the ability of this set-up to perform laboratory-based dynamic imaging, specifically in small animal models, and with the possibility of longitudinal studies.

## Introduction

For many years, X-ray imaging has been an important part of both diagnostic medical imaging and non-destructive sample characterisation, including quality assurance, security checks and material research. One of the advantages of X-ray imaging compared to other modalities, for example magnetic resonance imaging (MRI), is that an image can be captured very quickly, which facilitates capture and improved understanding of dynamic processes. Dynamic imaging sequences can improve medical diagnostics, help biomedical researchers to better understand physiology, and capture treatment effects in real time. Real-time image sequences are utilised clinically in fluoroscopy to examine dynamic processes, but it can be difficult to detect soft tissue structures using only conventional attenuation-based X-ray imaging.

To achieve better soft tissue contrast, it is useful to exploit the X-ray phase properties of the sample. These phase effects can be described using the complex refractive index *n* = 1 − *δ* + i*β* at a given X-ray energy, where *β* is related to the linear attenuation coefficient *μ,* and *δ* describes the phase shift. For hard X-rays and materials with low atomic numbers like biological tissues, *δ* is typically two to three orders of magnitudes higher than *β*, which provides enhanced contrast in phase-contrast X-ray imaging (PCXI). The strong difference in phase properties between tissue and air results in excellent phase contrast, making PCXI ideally suited to respiratory imaging. In recent years, several X-ray phase-contrast imaging methods have been developed^1^, including the X-ray grating interferometer^2,3^, propagation-based phase-contrast X-ray imaging (PB-PCXI)^4^ and the edge illumination X-ray phase-contrast technique^5,6^.

Short exposure times are essential in dynamic imaging to visualise a moving sample or detect changes in the sample over time. Some phase-contrast techniques require several exposures to extract phase contrast^2,3,7^, and so in this manuscript we focus on a technique where only a single exposure is necessary, PB-PCXI. Another advantage of this method is the simplicity of the set-up, which does not require any additional optical elements with respect to a conventional attenuation-based X-ray imaging set-up. Those elements dramatically reduce the flux reaching the detector, extending exposure times and making dynamic *in vivo* imaging more difficult. PB-PCXI requires only a partially coherent X-ray beam and a significant sample-detector distance, in this case 1 m, but in general, dependent on the sample, source properties (energy spectrum, source size, beam divergence) and the desired resolution. As a consequence of the coherence requirements, PB-PCXI has so far been limited to synchrotron facilities and laboratories with micro-focus X-ray tubes^8,9^. Dynamic or high-speed PCXI is usually only performed at synchrotron facilities^10^, since the small source size of micro-focus x-ray tubes limit the available flux.

Several efforts are currently underway that aim to fill the gap between synchrotrons and conventional X-ray sources with powerful laboratory X-ray sources, like the Tsinghua Thomson Scattering X-ray Source (TTX) in China^11^, ThomX in France^12^, cERL in Japan^13^ and the liquid-metal-jet source from Excillum in Sweden^14^. One of these is the Munich Compact Light Source (MuCLS), developed by Lyncean Technologies^15^, which provides a partially spatially coherent, low divergence, quasi-monochromatic X-ray beam in a laboratory environment. The X-rays are produced by colliding an intense laser beam with a counter-propagating electron beam, which revolves around a small electron storage ring. By employing inverse Compton scattering instead of permanent magnet undulators, it is possible to shrink the storage ring to a few meters in circumference, and to generate X-rays that are tunable between 15 keV and 35 keV^16–18^. The divergence of the beam is defined by a fixed aperture with an opening angle of 4 mrad divergence, which leads to a slightly increased bandwidth of about 1% if changing the energy from 15 keV to 35 keV^17^. The divergence is sufficiently low to enable long sample-to-detector distances (see numerical argument in Gradl *et al*.^18^), and high flux (up to 3 × 10^10^ photons/sec), both of which are vital for capturing dynamics using PB-PCXI. Furthermore, the low divergence of the X-ray beam leads to a beam diameter of about 16 mm at a distance of 4 m away from the X-ray source, where a set-up for PB-PCXI is located. This X-ray beam can fully illuminate a mouse lung, avoiding the need to scan such a sample through the beam. The X-ray flux density provided by the MuCLS allows the capture of low noise lung images at exposure times as low as 50 ms^18^, minimizing any motion blur caused by breathing.

*In vivo* dynamic respiratory phase-contrast X-ray imaging has the potential to greatly increase physiological understanding and accelerate new therapies towards the clinics. Examples to date include advances in neonatal ventilation strategies^19^ and assessment of new treatments for cystic fibrosis airway disease^20^. The results we present here demonstrate that with the proper animal imaging set-up^21^, quantitative time-resolved respiratory imaging is possible at a laboratory source based on inverse Compton scattering, and is thus no longer limited to large-scale synchrotron facilities.

## Results

### Lung motion

**Figure 1:**
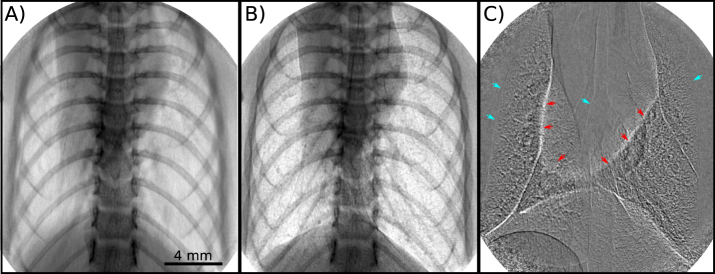
Propagation-based X-ray phase-contrast images of a mouse chest *in vivo* show the increase in image quality that is possible with sufficient flux to image the lungs without motion blur. In panel (A), an image was captured mimicking the exposure conditions at low-flux X-ray sources, resulting in motion blur due to the required averaging over breath cycles (here: one cycle during the 500 ms exposure). Panel (B) shows the benefit of a high flux source, which enables short exposures during plateaus of mechanical ventilation, in this case a 200 ms exposure triggered at the beginning of a 222 ms peak-inspiration breath-hold. Panel (C) reveals detailed structures in the lungs via a pseudo-differential image, achieved by subtracting sequential 200 ms breath-hold exposures between which there is a slight movement of the lungs and negligible movement of the skeleton. The red arrows highlight the border of the heart and the blue the edges of the lung and airways. The resulting structure seen within the lungs includes the small airways that are not easily seen in Panel (B).

During propagation-based X-ray phase-contrast imaging of the lungs, the air sacs in the lungs act like a large number of lenses, focusing the X-ray light into a speckle pattern^22^. Until now, most efforts to image the lungs outside a synchrotron required exposure times that resulted in lung images blurred by motion. In this case, speckles or airway structures are not resolved, as seen in Fig. 1A, where the exposure time is equal to the breath period. With the flux density provided by the MuCLS, however, it is possible to use a short enough exposure to avoid motion blur. In addition, a small-animal ventilator can be used to provide a sustained inflation, with a trigger sent to the detector in order to capture an image of the lung at the same point of each breath cycle (Fig. 1B), enabling monitoring over an extended period. Compared to Fig. 1A, Fig. 1B shows sharper edges of the lung and renders visible the characteristic speckle structure that results from multiple scattering by the alveoli. Triggering an image at the same point of each breath cycle is useful for imaging treatment delivery to the lungs^23^, monitoring the lung condition over time, or capturing an *in vivo* CT where the lungs must be at the same inflation level for each angular projection^24,25^. If, however, the lungs move slightly between two exposures, but the skeletal system is in the same position, the difference between the two images will reveal intricate lung structures in a pseudo-differential contrast, as shown in Fig. 1C.

**Figure 2:**
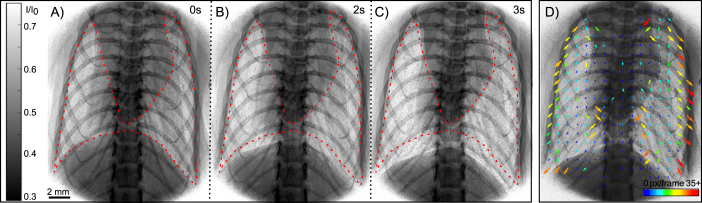
The motion of murine lungs during a slow breath cycle is imaged *in vivo* using propagation-based X-ray phase-contrast imaging. The red dotted outline, fixed at the start of the breath, highlights the changes in the shape of the lung during inhalation. Panels (A–C) show the first 3 s of the whole breath cycle (8 s). The expansion of the lung and increase in image intensity during inhalation is clearly visible. Supplementary Video S1 shows several breaths, captured with 15 images per breath. Panel (D) shows the corresponding X-ray velocimetry map during inhalation. As predicted, the largest movement is located at the lung periphery, as indicated by the warm end of the spectrum colour map used to display vector length. The maximum displacement vector length in (D) is 56 px.

The speckle pattern seen from the lungs can also be tracked over time using X-ray Velocimetry (XV) to map the motion of each part of the lungs and identify any regions that are not moving as expected^24^. This can help with diagnosis and monitoring of lung diseases including emphysema, lung cancer and cystic fibrosis^26^. Lung motion measurements are also useful in understanding lung tissue oscillations in response to different ventilation frequencies^27^ and to map local airflow for the design of drug delivery systems^28^. Figure 2A–C shows three points within the breath cycle, with the periphery of the lungs at full exhalation shown in red. The edge-enhancement induced by the phase-shifts clearly defines the edge of the lungs, and as such the increase in lung volume during inhalation can clearly be seen. XV of the lung expansion and contraction throughout the breath can reveal more local information, as displayed in Fig. 2D. The vectors are oriented pointing downwards and outwards, consistent with lung motion during inhalation, with the largest displacement seen at the edges of the lungs. This is attributed to the large physical displacement of the outer edges of the lungs as inflation of the inner- and mid-alveoli expands the lung tissue. As expected, the smallest displacement of lung tissue is observable in the center where the central airway tree is located.

### Nasal treatment delivery

Respiratory treatment delivery is difficult to capture *in vivo* and regional deposition is difficult to predict^29^. Liquid instillation to the mouse respiratory system via the nose is a primary route of administration for treating respiratory diseases (e.g. gene vector delivery to correct Cystic Fibrosis airway surface dysfunction^30^), for modeling lung disease by instilling debris or agents that damage the airways/lung^31^, and for testing the toxicity of substances that may enter the lungs^32^. In humans, nasal instillation can also be used as a way to administer pediatric anaesthetic^33^. We show here that we can non-invasively visualize the dynamics of liquid distribution during and immediately after delivery into one nostril of the mouse nose. This is particularly relevant for airway gene transfer research in mouse models which use a bolus fluid dosing into the nose or trachea, leaving the second nostril as a control^30^. Although studies of nasal fluid dose delivery have been performed at a synchrotron^34^, the present study represents the first time live non-invasive imaging of a nasal liquid distribution has been performed at a laboratory source.

Nasal fluid dosing was performed by placing the tip of a micro-syringe just above the nose, with delivery controlled using an infusion pump with a remote driver in order to image during treatment. For this first proof of principle study, we used a Sky-Blue/iodine mixture and delivered the fluid using a range of different volumes and rates, with various settings leading to different distributions in the murine airways. At the beginning of liquid delivery, a small droplet formed at the tip of the syringe, and was then inhaled spontaneously by the mouse. The phase-contrast renders the airways edges visible and the iodine is highly-absorbing so the location of the treatment can be seen even when overlaid with the dense bones of the skull. Sky-Blue is used as a fluorescent dye to study treatment deposition after excising the lungs, which means that future studies could spatially correlate the *in vivo* X-ray measurements with proven and standard *ex vivo* fluorescence measurements post-experiment^35,36^.

**Figure 3:**
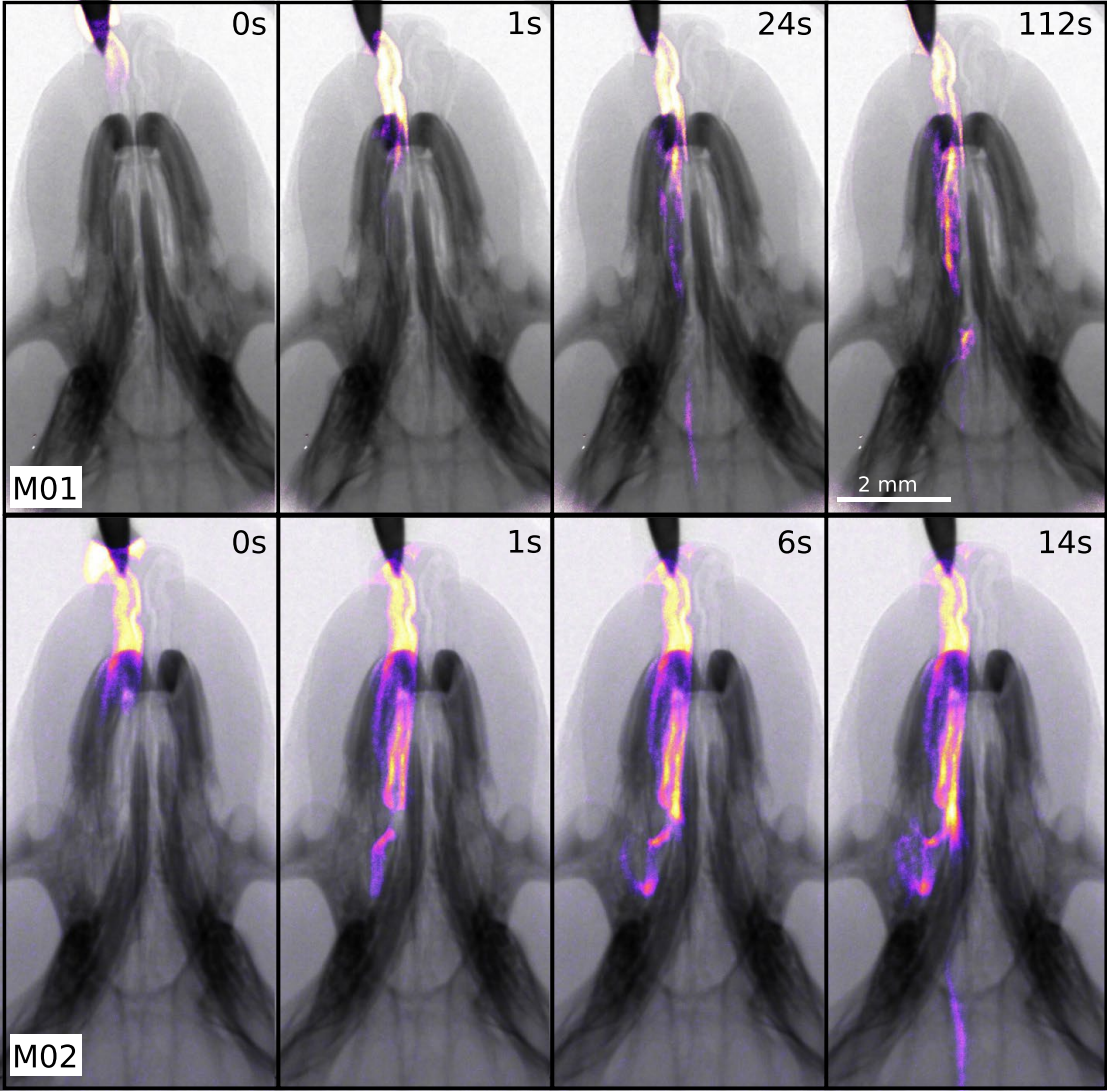
Delivery of liquid instillations to the nose of a mouse. The first row shows four time points during a relatively slow delivery of 20 µl of fluid over 500 s into one nostril of mouse M01. The second row shows the faster delivery, 40 µl in 250 s (mouse M02). The pseudo-coloured images show the fluid distribution. The origin of time axis was chosen at the point in time when the mouse inhaled the first liquid droplet.

Figure 3 shows frames from the time series of two different mice. The mice were breathing spontaneously and were not connected to the ventilator. Mouse M01 received a 20 µl fluid delivery in 500 s, and mouse M02 a 40 µl volume in 250 s. The pseudo-coloured images highlight the location of the contrast fluid. We successfully observed the distribution of the liquid over time, with the slower delivery draining down the side of the pharynx (Fig. 3, upper row) towards the trachea and the faster delivery pooling in the sinus and moving down the pharynx (Fig. 3, lower row). This kind of imaging could be used to predict the volume and timing of deposition in different locations, given a particular speed and volume of fluid delivery^23,34^. The dynamics of the deliveries are shown in Supplementary Videos S2A and S2B.

### Mucociliary Transport

A third application of this PB-PCXI respiratory imaging set-up is the quantification of mucociliary transport (MCT). Inhaled pathogens and particulates are normally cleared from the lungs along the airway surfaces and towards the mouth by the coordinated beating of cilia, microscopic hair-like structures present in the airway lining fluid. This process is an essential mechanism for maintaining healthy lungs, but MCT is inhibited in some conditions such as Primary Ciliary Dyskinesia and Cystic Fibrosis^37^. The ability of the MCT system to clear debris can be used as an indicator of airway health. Bulk clearance by MCT is typically assessed *in vitro* using dye and optical imaging methods applied to excised airway tissue or air liquid interface cultures^38,39^. To date, the only method by which individual particle MCT has been measured *in vivo* is with X-ray imaging^40,41^. The ability to non-invasively quantify MCT using X-rays means these measurements can be made following delivery of treatments to assess treatment efficacy^42^.

**Figure 4:**
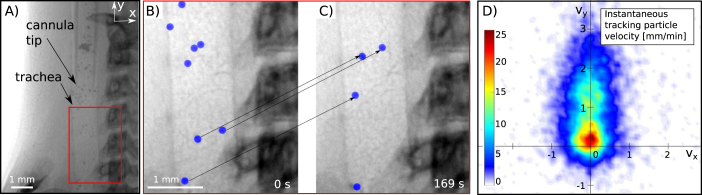
(A) Mucociliary clearance of inhaled beads in the trachea. Panels (B,C) show a magnified image of the trachea at different time-points with tracked beads marked in blue by the analysis software. Panel (D) shows the probability heat-map of the particle velocity due to the motion of the mucus layer. Positive *v*_*y*_ direction: Transport towards the larynx. The colourbar indicates the number of tracked steps in the given velocity bin.

Time-sequence imaging of the transit of deposited MCT tracking beads was performed in the trachea of a recently deceased mouse. As mucociliary transport continues for at least an hour after death, this experiment is able to show the experimental viability of particle tracking experiments at compact light sources like the MuCLS. Before imaging, high refractive index glass beads (Corpuscular, 78 µm diameter) were delivered into the trachea of the mouse with a dry powder insufflator (Penncentury, Model DP-04). These beads act as inhaled debris, and can be observed and tracked as they are cleared along the trachea of the mouse^42,43^. Figure 4A shows a side-view of the trachea including the cannula via which the beads were delivered. The red box highlights the magnified region shown in panels (B) and (C) at two different time points. The blue dots mark the location of beads, labeled by the software used to quantify clearance speeds^43^. The movement of the beads over a significant distance (1.5 mm) is clearly visible in these two images and also in the Supplementary Video S3 found in the supplementary material for Fig. 4. A modified version of the bead tracking algorithm described by Donnelley *et al*.^43^ was able to detect and track the particles. The result of the instantaneous tracking particle velocity in mm/min is shown in panel (D). A total of 6720 tracked particle steps is shown, with velocity direction corresponding to the same orientation/axes as in Fig. 4A (i.e. the positive *v*_*y*_-axis indicates movement towards the mouth of the mouse). A particle that was not moving would contribute to the counts at the origin. The resulting particle velocities, between 0–3 mm/min and directed towards the mouth, are consistent with published data^42,44^. Since the airway clearance mechanism transports the beads slowly we used an exposure time of 500 ms to increase the signal-to-noise ratio (SNR) to facilitate automatic bead detection.

## Discussion

We have presented *in vivo* dynamic imaging experiments with mice using propagation-based X-ray phase-contrast within a laboratory. While studies like this have until now been limited to synchrotron facilities, we have shown here that the MuCLS can produce high-visibility propagation-based phase-contrast images, sufficient to track the respiratory processes described in this paper.

Here we make a brief comparison between the MuCLS and synchrotron sources, looking at some of the key experimental parameters necessary for dynamic phase-contrast x-ray imaging. To produce high contrast phase-contrast fringes, high spatial coherence and a narrow energy bandwidth is required. The spatial coherence at the MuCLS^17^, 5 m away from the source (horizontal source size is about 45 µm (RMS)), is around 26% of that seen at modern dedicated medical imaging beamlines, like the Australian Synchrotron 138 m Imaging and Medical Beamline (IMBL) built in 2013, where the horizontal source size is about 320 µm (RMS)^45^. While the energy bandwidth is wider at the MuCLS than at synchrotrons (≈4% compared to ≈0.1% at the IMBL^46^), this is still a much narrower energy range than produced by conventional X-ray sources. In addition, PB-PCXI is quite immune to polychromaticity in this range, with different energies serving only to broaden or narrow the central fringe a little^8^. Penumbral blurring of around 10 µm RMS for the experimental set-up used here is perhaps the most detrimental effect compared to synchrotron imaging, approximately four times the extent found at the IMBL for 1 m propagation. However, this value will depend also on the chosen propagation distance.

Dynamic biomedical imaging requires short exposure times to avoid motion blur. Although the flux is not as high as that seen from a synchrotron source, the exposure times are sufficiently short to capture respiratory dynamics, treatment response and other biological motion (with the exception of the heartbeat). Since the data was acquired, an upgrade of the source has been implemented, which increases the X-ray flux up to a factor of 2 compared to the initial parameters given in Eggl *et al*.^17^. The increased flux can provide either higher SNR or permit the use of shorter exposure times. The exposure times we have utilized at the MuCLS for murine lung imaging (e.g. Fig. 1B, 200 ms) are indeed already comparable to the exposure times used for synchrotron murine lung imaging (e.g. 100 ms at beamline BL20B2 at the SPring-8 synchrotron in Donnelley *et al*.^23^, noting that the use of a different detector means it is difficult to compare quantitatively).

However, the imaging speed does not only depend on the available flux, but also on the read-out and saving time of the detector, which is non-negligible at these frame rates. With respect to the results presented here, lung motion imaging could be performed at a faster breathing rate if the read-out/saving time was reduced (currently limited to 2.1 fps with 200 ms exposures). These higher frame rates are also required for four-dimensional phase-contrast X-ray studies that use high speed tomographic imaging to capture dynamics^47^, e.g. a fly beating its wings^48^ or bread baking^49^, which have so far been performed only at high flux synchrotron facilities.

The results presented here show that it is possible, using phase-contrast imaging at a laser-undulator based compact X-ray source, to capture and quantify measures of respiratory health. The three respiratory processes captured, mucociliary clearance velocity, lung motion and drug delivery, are focused around treatment development in small animal models. The ease of access provided by a laboratory source enables longitudinal studies to measure disease development or treatment response over weeks, which can be difficult at synchrotron sources where experimental access is limited. Finally, these dynamic imaging capabilities are by no means limited to respiratory imaging, and there are a range of biological and industrial applications that could benefit from this set-up.

## Methods

### Imaging set-up

Imaging was performed at the Munich Compact Light Source. The source was delivered by Lyncean Technologies, Inc.^15^. The Imaging beamlines were set up by TUM. Detailed descriptions of the working principles of the MuCLS are published in several previous publications^16,17,50^.

**Figure 5:**
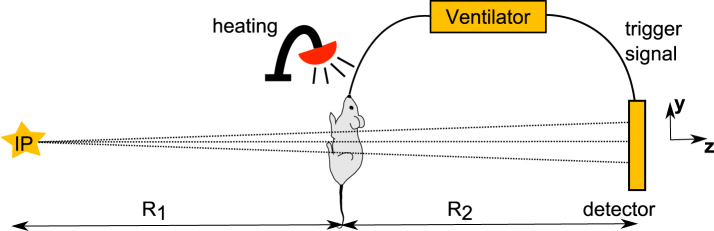
Set-up for *in vivo* mouse imaging at the Munich Compact Light Source. The mouse is held head-high in a vertical position to enable either an anteroposterior (AP) projection (e.g. of the lungs) or a lateral (Lat) projection (e.g. through the trachea). The mouse can be intubated and ventilated, with the ventilator configured to send a trigger signal to the detector to minimize respiratory motion during an exposure of the lungs. The source-to-sample distance *R*_1_ was 4 m and the propagation distance *R*_2_ was chosen as 1 m.

For all experiments, quasi-monochromatic X-rays at 25 keV were chosen with a flux up to 0.7 × 10^10^ ph/s. Figure 5 shows a sketch of the installed set-up for *in vivo* imaging. The interaction point (IP), where laser beam and electron beam collide, forms the source of the X-rays. The source-to-sample distance R_1_ can be set within 3.5 m to 5 m, and the sample-to-detector distance R_2_ can be adjusted from a few mm to 1.3 m. In the experiments presented here, R_1_ = 4 m and R_2_ = 1 m were used to visualize phase effects. The sample is located on a translation and rotation stage. At the sample position the beam size was approximately 16 mm in diameter. X-rays were converted to visible light with a 20 µm thick Gadox screen (Gd_2_O_2_S:Tb) which is deposited on a 2:1 fiber optic taper and coupled to an Andor Zyla 5.5 sCMOS camera. The sCMOS detector has an array size of 2560 × 2160 with a resulting detector pixel size of 13 µm. The projections obtained in this study were all corrected for beam profile and detector inhomogeneities, and for dark current. Furthermore, in some parts of the study a small animal ventilator (flexiVent FX, SCIREQ) was used to send a trigger signal to the detector, so that an image is always taken at the same point of the breath cycle. We estimate that this set-up results in an entrance surface dose rate of 0.35 mGy per 100 ms exposure.

### Animal ethics statement

All procedures for animal handling and experiments were performed in accordance with protocols approved by the Regierung von Oberbayern (District Government of Upper Bavaria; Approval no: 55.2-1-54-2532-108-13). The experimental group consisted of 5 animals. Mice were kept in isolated ventilated cages (IVC-Racks; BioZone, Margate) supplied with filtered air in a 12-hr light/12-hr dark cycle (lights on from 06:00–18:00). Food (standard chow) and water were available *ad libitum*.

### Animal handling

C57BL/6 mice (age 8–14 weeks, female, weight 18–22 g) were anaesthetized by intraperitoneal injection of a mixture of Medetomidine (0.5 mg/kg body mass), Midazolam (5.0 mg/kg body mass) and Fentanyl (0.05 mg/kg body mass). Except for nasal applications, the animals were then intubated by a non-surgical technique (Brown *et al*. 1999) with a 20 Ga cannula. Then, the mouse was placed into the radiation-shielded imaging hutch where it was kept warm with a heater and ventilated in some cases (flexiVent FX, SCIREQ). Immediately after imaging was completed, the still-anesthetized mice were humanely killed by exsanguination.

### Lung motion

Figure 1 shows the difference between an image taken over a whole breath cycle (A) and a short exposure ventilator-triggered image (B). For the image shown in Fig. 1A, the ventilator was set to 250 ms inspiration and 250 ms expiration and an exposure time of 500 ms was used. For the image shown in Fig. 1B, the ventilator was set to 222 ms inspiration, 222 ms breath-hold and 222 ms expiration. The ventilator sent a trigger signal to the detector at the beginning of the breath-hold. This kind of breath-hold ventilation pattern is used in mice for CT imaging^51^. An image was then captured at each breath-cycle with a 200 ms exposure time. Both images were taken with the same mouse.

X-ray Velocimetry (XV) was performed on a lung motion sequence. The speckles generated by the alveoli are tracked over the breath and their movement is transformed to a vector. Exposure times of 200 ms and a decreased breathing rate were used to capture these images. The mouse was ventilated with 120 breaths/min (Tidal volume 0.6 mL), then the breathing rate was reduced to 8 breaths/min for 5 breaths of imaging. Over one slow breath cycle, 15 images were captured. This imaging protocol was chosen following the parameters used by Murrie *et al*.^52^. A 5 px median filter and a 20–150 px bandpass frequency filter were applied to the images to reduce image noise and enhance the visibility of the lung speckles. A mask was applied to the images to ensure only the lung tissue underwent analysis. Analysis windows, placed every 8 px in both the *x* and *y* directions, calculated a displacement vector based on speckle movement. An iterative approach was used, where larger windows gave an estimate of the displacement, and further passes with increasingly smaller windows refined vector directions and lengths. The final pass was performed using 64 × 64 pixel windows.

### Nasal and pulmonary instillation

A 1:9 mixture of iodine-based contrast agent (Ultravist-370, 370 mg Iodine/ml) and Sky-Blue (a contrast agent for fluorescence microscopy, Excitation/Emission = 670 nm/710 nm; Kisker Biotech, 10 mg/ml) was loaded into a 100 µl Hamilton syringe in a WPI UltraMicroPump UMP3, remotely controlled from outside the imaging hutch using a WPI Ultra Micro4 controller. The fluid is a surrogate for a typical respiratory treatment delivery, and was spontaneously inhaled via the nose by the mouse. Images were captured at 2 Hz, using an exposure time of 100 ms. The pseudo-coloured images highlight the location of the contrast fluid. These coloured areas were created by taking the difference in intensity between the current and pre-delivery image frame, similar to the analysis performed in Donnelley *et al*.^34^. Images were aligned before colour coding via a cross-correlation based image registration.

### Mucociliary Transport

This experiment was performed on a recently deceased mouse. Before imaging, high refractive index glass beads (Corpuscular, 78 µm diameter) were delivered into the trachea of the mouse with a dry powder insufflator (Penncentury, Model DP-04). An exposure time of 500 ms was used with a frame rate of 1.15 fps. Image analysis was performed in Matlab R2017a (The Mathworks, Natick, USA). Intensity-based automatic image registration, performed using the Matlab imregister command, was used to register each image to the first frame to correct for translations throughout the sequence. Particle tracking and motion analysis (histogram production) were performed using a previously described algorithm^43^, with two modifications. Firstly, a mask was applied to all images to only detect particles within the trachea. Secondly, due to the much smaller apparent size of the beads, the published algorithm performed poorly because the beads were not detected well by the circular Hough transform algorithm. This code was therefore replaced by an intensity thresholding and size exclusion algorithm set to identify dark objects with a size range matching the beads.

## Electronic supplementary material

Supplementary Video 1

Supplementary Video 2A

Supplementary Video 2B

Supplementary Video 3
